# Vaccination Coverage Among Children Aged 2 Years — U.S. Affiliated Pacific Islands, April–October, 2016

**DOI:** 10.15585/mmwr.mm6720a3

**Published:** 2018-05-25

**Authors:** Ashley Tippins, Neil Murthy, Mehreen Meghani, Amy Solsman, Carter Apaisam, Merlyn Basilius, Maribeth Eckert, Peter Judicpa, Yolanda Masunu, Kelsey Pistotnik, Daisy Pedro, Jeremy Sasamoto, J. Michael Underwood

**Affiliations:** ^1^Immunization Services Division, National Center for Immunization and Respiratory Diseases, CDC; ^2^Epidemic Intelligence Service, CDC; ^3^Leidos, Inc., Atlanta, Georgia; ^4^Federated States of Micronesia Department of Health, Education and Social Affairs; ^5^Republic of Palau Ministry of Health; ^6^American Samoa Department of Public Health; ^7^Republic of the Marshall Islands Ministry of Health and Human Services; ^8^Commonwealth of the Northern Mariana Islands Commonwealth Healthcare Corporation.

Vaccine-preventable diseases (VPDs) cause substantial morbidity and mortality in the United States Affiliated Pacific Islands (USAPI).* CDC collaborates with USAPI immunization programs to monitor vaccination coverage. In 2016, ^†^ USAPI immunization programs and CDC piloted a method for estimating up-to-date status among children aged 2 years using medical record abstraction to ascertain regional vaccination coverage. This was the first concurrent assessment of childhood vaccination coverage across five USAPI jurisdictions (American Samoa; Chuuk State, Federated States of Micronesia [FSM]; Commonwealth of the Northern Mariana Islands [CNMI]; Republic of the Marshall Islands [RMI]; and Republic of Palau).^§^ Differences in vaccination coverage between main and outer islands^¶^ were assessed for two jurisdictions where data were adequate.** Series coverage in this report includes the following doses of vaccines: ≥4 doses of diphtheria and tetanus toxoids and acellular pertussis vaccine (DTaP); ≥3 doses of inactivated poliovirus vaccine (IPV); ≥1 dose of measles, mumps, and rubella vaccine (MMR); ≥3 doses of *Haemophilus influenzae* type B (Hib) vaccine; ≥3 doses of hepatitis B (HepB) vaccine; and ≥4 doses of pneumococcal conjugate vaccine (PCV); i.e., 4:3:1:3:3:4. Coverage with ≥3 doses of rotavirus vaccine was also assessed. Completion of the recommended series of each of these vaccines^††^ was <90% in all jurisdictions except Palau. Coverage with the full recommended six-vaccine series (4:3:1:3:3:4) ranged from 19.5% (Chuuk) to 69.1% (Palau). In RMI and Chuuk, coverage was lower in the outer islands than in the main islands for most vaccines, with differences ranging from 0.9 to 66.8 percentage points. Medical record abstraction enabled rapid vaccination coverage assessment and timely dissemination of results to guide programmatic decision-making. Effectively monitoring vaccination coverage, coupled with implementation of data-driven interventions, is essential to maintain protection from VPD outbreaks in the region and the mainland United States.

USAPI immunization program staff members report that the geographic remoteness of the USAPI (Figure), particularly the outer islands, affects vaccine distribution and delivery, strains limited resources, and adversely affects vaccination coverage. Additional challenges to maintaining adequate vaccination coverage include a high prevalence of socioeconomic disparities, inaccessibility of vaccination providers and clinics, and difficulty tracking highly mobile populations (*1*). The United States maintains a military presence in the region, and USAPI citizens can travel, live, and work in the United States without restriction.^§§^ As a result of frequent travel, VPD outbreaks in the USAPI have been associated with importations and outbreaks in the mainland United States (*2*,*3*). The USAPI receive economic assistance, immunization infrastructure support, and limited vaccines through Section 317 of the Public Health Services Act, as well as technical assistance from CDC, to ensure protection of the population from VPDs (*4*).

**FIGURE:**
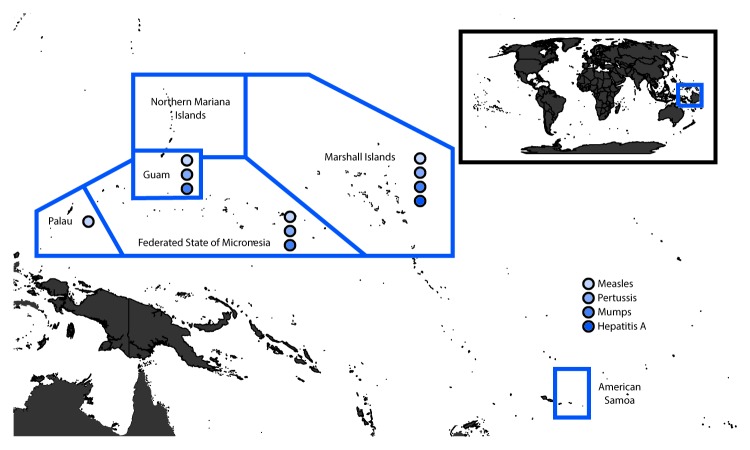
Vaccine-preventable disease outbreaks — U.S. Affiliated Pacific Islands, 2002–2018* * Federated States of Micronesia: measles 2014, pertussis 2007, mumps 2009 and 2017–2018; Guam: measles 2002–2004 and 2013, pertussis 2001–2006 and 2009–2010, mumps 2006 and 2010–2011; Palau: measles 2003; Marshall Islands: measles 2003, pertussis 2009, mumps 2017, hepatitis A 2017.

Demographic and vaccination data were collected for children aged 24–35 months (2 years) at the time of data collection in each USAPI. Data sources included labor and delivery log books, vital statistics birth rosters, medical records, public health vaccination log books, and electronic records from the resource and patient management records system and the immunization information system, where available. Up-to-date vaccination status (number of children who received the number of doses recommended by age 24 months, among all children identified by health records) was estimated according to recommended vaccination schedules, which vary across the USAPI.

Unique identifiers, including each child’s name, sex, date of birth, geographic region, country of birth, name of parent(s), type of vaccine administered, and date of vaccine administration were abstracted from available records in each jurisdiction. Data collected from each source were matched, deduplicated, and merged to create a complete vaccination record for each child. Geographic differences in vaccination coverage between main islands and outer islands were assessed for Chuuk and RMI.^¶¶^

## Jurisdictional Childhood Vaccination Coverage

Hepatitis B vaccine birth dose*** coverage exceeded 85%^†††^ for each USAPI, except Chuuk (53.5%) (Table 1). Coverage for all other routinely recommended vaccines fell below jurisdictional targets of 90%, except in Palau, where coverage with ≥3 doses of IPV, ≥3 doses of Hib, and ≥3 doses of HepB was 94.6%, 93.1%, and 93.1%, respectively. Palau also had the highest 4:3:1:3:3:4 coverage (69.1%); coverage was <50% in American Samoa (47.9%), RMI (43.0%), CNMI (40.0%), and Chuuk (19.5%).

**TABLE 1:** Estimated vaccination coverage among children aged 24–35 months,* by selected vaccines and doses — United States Affiliated Pacific Islands, 2016

**% Vaccination coverage**					
**Vaccine**	**Chuuk, FSM**	**Republic of the Marshall Islands**	**Republic of Palau**	**Commonwealth of the Northern Mariana Islands**	**American Samoa**
	**(N = 1,218)**	**(N = 1,312)**	**(N = 259)**	**(N = 1,140)**	**(N = 1,180)**
**DTaP**					
≥3 doses	71.6	72.0	94.6	59.5	84.8
≥4 doses	36.7	54.7	79.9	44.7	62.9
**IPV** (≥3 doses)	71.2	72.7	94.6	58.9	82.8
**MMR**					
≥1 dose	88.4	68.7	85.7	57.9	75.5
≥2 doses^†^	68.9	51.0	76.8	NA	NA
**Hib** (≥3 doses)	53.7	63.5	93.1	48.7	63.1
**HepB**					
Birth dose^§^	53.5^¶^	86.7^¶^	96.6^¶^	97.5**	96.7^¶^
≥3 doses	77.8	76.0	93.1	62.1	82.0
**PCV**					
≥3 doses	51.0	68.3	87.3	58.0	78.8
≥4 doses	22.2	46.7	70.7	42.4	61.5
**Rotavirus** (≥3 doses)	16.8	46.5	81.9	40.0	NA^††^
**Combined series** ^§§^	19.5	43.0	69.1	40.0	47.9

A**bbreviations:** CNMI = Commonwealth of the Northern Mariana Islands; DTaP = diphtheria and tetanus toxoids and acellular pertussis vaccine; FSM = Federated States of Micronesia; HepB = hepatitis B vaccine; Hib = *Haemophilus influenzae* type B vaccine; IPV = inactivated poliovirus vaccine; MMR = measles, mumps, and rubella vaccine; NA = not applicable; PCV = pneumococcal conjugate vaccine; RMI = Republic of the Marshall Islands.

* Children were aged 2 years at time of data collection: Chuuk, FSM: April 2016; RMI: June 2016; Palau/CNMI: August 2016; American Samoa: October 2016.

^†^ CNMI and American Samoa recommend the second dose of MMR at age 4–6 years.

^§^ One dose of HepB administered within 3 calendar days of birth.

^¶^ Only includes children born in the jurisdiction: Chuuk, FSM (n = 1,149); RMI (n = 1,181); Palau (n = 237); American Samoa (n = 1,083).

** Includes all children aged 2 years; data insufficient to identify children born outside CNMI.

^††^ Rotavirus vaccine is not routinely administered in American Samoa.

^§§^ The combined six-vaccine series (4:3:1:3:3:4) includes ≥4 DTaP doses, ≥3 IPV doses, ≥1 MMR dose, ≥3 Hib doses, ≥3 HepB doses, and ≥4 PCV doses.

Coverage with individual vaccines varied considerably across USAPI jurisdictions. For example, coverage with ≥3 doses of IPV ranged from 94.6% (Palau) to 58.9% (CNMI), and with ≥1 MMR dose from 88.4% (Chuuk) to 57.9% (CNMI). For all vaccines requiring more than 1 dose, coverage decreased with subsequent doses^§§§^; this decrease also varied by vaccine and jurisdiction. For example, coverage with ≥3 doses of DTaP ranged from 94.6% (Palau) to 59.5% (CNMI), and coverage with ≥4 doses of DTaP ranged from 79.9% (Palau) to 36.7% (Chuuk).

## Subjurisdictional Differences in Childhood Vaccination Coverage

Vaccination coverage was lower in the outer islands compared with that in the main islands in Chuuk and RMI for most vaccines (Table 2). In Chuuk, coverage with all vaccines except MMR was higher in the main island (Weno) than in the outer islands. Differences ranged from 10.1 to 30.6 percentage points for ≥3 doses of HepB and ≥4 doses of DTaP, respectively. In Ebeye, one of the two main RMI islands, coverage with all vaccines except HepB birth dose was 15.3–51.4 percentage points higher than that in Majuro, the other main island, and 25.3–66.8 percentage points higher than that in the outer islands. Similarly, coverage with all vaccines was higher in Majuro than coverage in the outer islands, except for ≥2 doses of MMR, ≥3 doses of Hib, and ≥3 doses of HepB. The largest disparity was in HepB birth dose coverage both in Chuuk, where there was a 35.8 percentage point difference between coverage in Weno (81.2%), and the outer islands (45.4%), and RMI, where coverage ranged from 94.9% in Majuro, to 88.9% in Ebeye, and 41.3% in the outer islands.

**TABLE 2:** Estimated vaccination coverage among children aged 24–35 months,* by selected vaccines and doses, and by main or outer island area† — Selected United States Affiliated Pacific Islands, 2016

Vaccine	Chuuk, FSM			Republic of the Marshall Islands^§^					
	Weno (main island)	Outer islands	Difference^¶^	Majuro (main island)	Ebeye (main island)	Outer islands	Difference (outer islands-Majuro)^¶^	Difference (outer islands-Ebeye)^¶^	Difference (Majuro-Ebeye)^¶^
	(N = 215)	(N = 1,003)		(N = 801)	(N = 275)	(N = 157)			
	%	%	%	%	%	%			
**DTaP**									
≥3 doses	80.9	69.6	-11.3	66.9	94.2	52.9	-14.0	-41.3	-27.3
≥4 doses	61.9	31.3	-30.6	46.4	89.8	33.8	-12.6	-56.0	-43.4
**IPV** (≥3 doses)	80.9	69.1	-11.8	68.3	94.2	51	-17.3	-43.2	-25.9
**MMR**									
≥1 dose	85.6	89	3.4	58.4	93.5	68.2	9.8	-25.3	-35.1
≥2 doses	69.8	68.9	-0.9	39.2	90.6	37.6	-1.6	-53.0	-51.4
**Hib** (≥3 doses)	69.8	50.3	-19.5	55.1	92	50.3	-4.8	-41.7	-36.9
**HepB**									
Birth dose**	81.2^††^	45.4^††^	-35.8	94.9^††^	88.9^††^	41.3^††^	-53.6	-47.6	6.0
≥3 doses	86.1	76	-10.1	70.5	94.6	63.1	-7.4	-31.5	-24.1
**PCV**									
≥3 doses	68.8	47.2	-21.6	66.9	82.2	46.5	-20.4	-35.7	-15.3
≥4 doses	39.5	18.4	-21.1	44.2	68	21	-23.2	-47.0	-23.8
**Rotavirus** (≥3 doses)	36.3	12.6	-23.7	45.6	73.8	7	-38.6	-66.8	-28.2
**Combined series^§§^**	36.7	15.9	-20.8	39	67.6	18.5	-20.5	-49.1	-28.6

**Abbreviations:** DTaP = diphtheria and tetanus toxoids and acellular pertussis vaccine; FSM = Federated States of Micronesia; HepB = hepatitis B vaccine; Hib = *Haemophilus influenzae* type B vaccine; IPV = inactivated poliovirus vaccine; MMR = measles, mumps, and rubella vaccine; PCV = pneumococcal conjugate vaccine; RMI = Republic of the Marshall Islands; USAPI = United States Affiliated Pacific Islands.

* Children were aged 2 years at time of data collection: Chuuk: April 2016; RMI: June 2016.

^†^ In some USAPI, population, government offices, health care facilities, and other services are centered on one or two “main islands.” Islands outside of the main island are referred to as “outer islands.” Outer islands can be located hundreds of miles away from the associated main island. Chuuk, FSM, and RMI both have main and outer islands.

^§^ Excludes children known to have lived in more than one local area in RMI (n = 79).

^¶^ Percentage point difference by local area.

** One dose HepB vaccine administered within 3 calendar days of birth.

^††^ Only includes children born in the jurisdiction: Chuuk, FSM (n = 1,149); RMI (n = 1,107; [Majuro n = 712; Ebeye n = 252; outer islands n = 143]).

^§§^ The combined six-vaccine series (4:3:1:3:3:4) includes ≥4 DTaP doses, ≥3 IPV doses, ≥1 MMR dose, ≥3 Hib doses, ≥3 HepB doses, and ≥4 PCV doses.

In Chuuk, coverage for the combined six-vaccine series was higher in Weno (36.7%) than that in the outer islands (15.9%). In RMI, Ebeye had the highest combined six-vaccine series coverage (67.6%), compared with Majuro (39.0%) and the outer islands (18.5%).

## Discussion

Vaccination coverage in the five USAPI assessed was lower than the national targets established by each jurisdiction and varied widely among children aged 2 years. Among these jurisdictions, only Palau met the coverage target of ≥90% for ≥3 doses of IPV, ≥3 doses of Hib, and ≥3 doses of HepB. Coverage with vaccine doses recommended in the second year of life, such as the fourth doses of DTaP and PCV, were substantially lower than coverage with doses recommended before the first birthday. The widespread prevalence of undervaccinated children in the USAPI allows for the rapid and recurrent spread of VPD outbreaks (*5*–*7*). Since 2000, at least 13 documented VPD outbreaks occurred in these islands, and importations to the United States are common (*2*,*3*).

Geographic differences in routinely recommended childhood vaccination coverage were identified in Chuuk and RMI, with substantially higher coverage with most vaccines documented among children on main islands than on outer islands. Proximity to health care providers on the main islands might contribute to these observed coverage differences. For example, HepB birth doses are normally administered in clinical settings at or shortly after the time of delivery, and the differences in birth dose coverage between main and outer islands might reflect differences in access to health care. Information on place of birth documented on medical records suggests that nearly 60% of children on outer islands are born at home and, therefore, might not have had an opportunity to receive a HepB birth dose at delivery. In Ebeye and Majuro, only 11% and <4% of births occur at home, respectively. Approximately 70% of outer island children were identified only by vaccination outreach logbooks, indicating that at the time of the assessment, they might have never accessed health care facilities on Majuro or Ebeye, where they would have been issued a medical record. In Chuuk, coverage with ≥1 dose of MMR was similar on the main island (86%) and outer islands (89%). This might be attributed to a 2014 mass MMR vaccination campaign, targeting all persons aged 6 months–49 years across the state, in response to a measles outbreak (*8*). Among children in this cohort who received at least one MMR dose (1,077), 42.2% received a dose during the 2014 campaign. Between the two main RMI islands, vaccination coverage was generally higher in Ebeye than in Majuro. Ebeye’s higher coverage might derive from its smaller population (9,614 according to 2011 census) and geographic size (0.12 sq. mi.) compared with that of Majuro (population = 27,797; area = 3.75 sq. mi.), which could facilitate Ebeye’s community outreach activities to target the entire population. These results underscore the importance of vaccination outreach in reducing coverage disparities between main and outer island children.

Before 2016, assessment of vaccination coverage was conducted infrequently because of the high cost and time commitment required to conduct household surveys. The results of this health facility–based assessment can serve as a baseline for coordinated USAPI and CDC programs to improve vaccination coverage. USAPI immunization programs and stakeholders are currently assessing a range of interventions to increase coverage in the region, including improving vaccine inventory management, eliminating missed vaccination opportunities, and establishing reminder and recall systems (particularly for doses recommended during the second year of life), in conjunction with improving communication and social mobilization measures to educate caregivers about the importance of additional vaccines beyond infancy (*9*,*10*). As a result of increased collaboration and ongoing engagement with CDC staff members and USAPI stakeholders, USAPI immunization programs are actively exploring or implementing these and other public health interventions to improve vaccination coverage in the region to reduce the occurrence of VPDs. For example, Palau is considering increasing the recommended number of well child visits to facilitate vaccination of eligible children; FSM is exploring methods to improve vaccine forecasting and ordering processes, as well as currently conducting catch-up vaccination campaigns in all states; and RMI is working with the Ministry of Health to increase immunization support staff members (Carter Apaisam, Federated States of Micronesia Department of Health, Education and Social Affairs; Merlyn Basilius, Republic of Palau Ministry of Health; Daisy Pedro, Republic of the Marshall Islands Ministry of Health; personal communications, April 2017).

The findings in this report are subject to at least four limitations. First, results might be subject to selection bias because children living in a jurisdiction who did not appear in any of the records that were collected might have been excluded from the analysis. Second, results might be subject to misclassification bias because children who have died or moved away from the jurisdiction but were not identified as such in existing records might be included in the analysis. A recent CDC assessment determined that 4% of persons targeted for a vaccination campaign in FSM using existing records were found to have moved away from the jurisdiction. Third, results might not be generalizable to all age cohorts because only children aged 2 years were assessed. Finally, because the USAPI jurisdictions recommend different vaccination schedules, jurisdictions might prioritize different vaccine targets, thereby making it difficult to compare coverage across the USAPI.

This was the first comprehensive assessment to measure childhood vaccination coverage concurrently across five USAPI. The record abstraction methodology enabled timely dissemination of results to decision makers, who were able to design, fund, and implement intervention strategies within a year of data dissemination in several of the USAPI. Continued and timely monitoring of vaccination coverage, coupled with implementation of vaccination outreach and other interventions, should remain a top priority for immunization programs to prevent future VPD outbreaks in the region and to prevent importation of cases to the mainland United States.

**Summary**

**What is already known about this topic?**

The United States Affiliated Pacific Islands (USAPI) face challenges because of their remoteness and limited resources. Low vaccination coverage has contributed to outbreaks in the USAPI; travel between the USAPI and the U.S. mainland has contributed to outbreaks of vaccine-preventable diseases (VPDs) on the mainland.

**What is added by this report?**

CDC piloted a method of estimating coverage by medical record abstraction in five USAPI jurisdictions. Coverage with the combined six-vaccine series by age 2 years ranged from 19.5% to 69.1%.

**What are the implications for public health practice?**

Record abstraction can help health authorities conduct surveillance, design and implement interventions, avoid VPD outbreaks, and reduce importation of cases to the mainland.

## Footnotes

* The USAPI consist of three territories or commonwealth nations (Guam, Commonwealth of the Northern Mariana Islands [CNMI], and American Samoa) and three freely associated sovereign nations (Federated States of Micronesia [FSM], Republic of the Marshall Islands [RMI], and Republic of Palau).

† Data were collected during the following months in 2016: Chuuk, FSM: April; RMI: June; Palau: August; CNMI: August; American Samoa: October.

§ Since 2014, biannual National Immunization Surveys (NIS) have been conducted in Guam to assess vaccination coverage among children aged 19–35 months. NIS has not been conducted in the other USAPI because a large proportion of households do not have telephones.

¶ In some USAPI, population, government offices, health care facilities, and other services are centered on one or two “main islands.” Islands outside of the main island are referred to as “outer islands.” Outer islands can be located hundreds of miles away from the associated main island.

** Differences in vaccination coverage between main and outer islands were assessed in Chuuk and RMI. Chuuk’s main island is Weno. RMI’s main islands are Majuro and Ebeye. Palau’s few outer islands are sparsely populated; child population size was not adequate to assess geographic differences, if any. Data were not adequate to classify geographic region reliably for children in CNMI or American Samoa.

†† Advisory Committee on Immunization Practices (ACIP) recommends routine administration of the second MMR dose at age 4–6 years, but states that the dose may be administered as early as age 13 months provided at least 4 weeks have elapsed since the first dose. FSM, Palau, and RMI recommend routine administration of the second dose as early as the minimum age of 13 months, provided the minimum interval has elapsed. These three jurisdictions do not routinely administer ACIP-recommended varicella vaccine or hepatitis A (HepA) vaccine; however, FSM and RMI recommend Bacillus Calmette-Guerin (BCG) vaccine at birth, which is not an ACIP-recommended vaccine. American Samoa does not routinely administer varicella vaccine or rotavirus vaccine. ACIP approves the use of several licensed Hib vaccine formulations; products used vary by jurisdiction. The Hib primary series includes receipt of ≥2 or ≥3 doses, depending on product type received; Hib full series includes primary series, and booster dose includes receipt of ≥3 or ≥4 doses, depending on product type received. Vaccine doses for all vaccines recommended by age 24 months are listed here, according to jurisdiction. Results in this report are limited to vaccines and number of doses common across all jurisdictions. *American Samoa*: ≥4 DTaP doses, ≥3 IPV doses, ≥1 MMR dose, ≥3 Hib doses, ≥3 HepB doses, and ≥4 PCV doses; *CNMI*: ≥4 DTaP doses, ≥3 IPV doses, ≥1 MMR dose, ≥4 Hib doses, ≥3 HepB doses, ≥4 PCV doses, ≥3 rotavirus vaccine doses, ≥2 HepA doses, and ≥1 varicella dose; *FSM*: 1 BCG dose, ≥4 DTaP doses, ≥3 IPV doses, ≥2 MMR doses, ≥3 Hib doses, ≥3 HepB doses, and ≥4 PCV doses; *RMI*: 1 BCG dose, ≥4 DTaP doses, ≥3 IPV doses, ≥2 MMR doses, ≥3 Hib doses, ≥3 HepB doses, and ≥4 PCV doses; *Palau*: 1 BCG dose, ≥4 DTaP doses, ≥3 IPV doses, ≥2 MMR doses, ≥4 Hib doses, ≥3 HepB doses, and ≥4 PCV doses.

§§ For more details, refer to “U.S. Affiliated Pacific Basin Jurisdictions: Legal, Geographic and Demographic Information” https://www.ruralhealthinfo.org/resources/2065.

¶¶ Differences in vaccination coverage between main and outer islands were assessed in Chuuk, FSM, and RMI. The main island in Chuuk is Weno. RMI has two main islands: Majuro and Ebeye. Palau’s few outer islands are sparsely populated; child population size was not adequate to report vaccination coverage by region. Data were not adequate to classify geographic region for children in CNMI or American Samoa reliably.

*** One HepB birth dose administered ≤3 calendar days from birth. Children were excluded from birth dose calculations if they were known to have been born outside of the jurisdiction. RMI results also exclude children with an unknown birth location.

††† HepB birth dose target (≥85%), individual vaccine targets (≥90%), and vaccine series targets (≥85%) set by the USAPI jurisdictions apply to children 19–35 months; children in this assessment were aged 24–35 months.

§§§ Data not shown for HepB, Hib, IPV, or rotavirus vaccine.
